# Applying Computational Engineering Modeling to Analyze the Social Impact of Conflict and Violent Events

**DOI:** 10.3390/e27101003

**Published:** 2025-09-26

**Authors:** Felix Schwebel, Sebastian Meynen, Manuel García-Herranz

**Affiliations:** 1 HWI, Ulmenliet 20, University of Hamburg/Hamburg University of Applied Sciences, 21033 Hamburg, Germany; 2 UNICEF, 3 United Nations Plaza, New York, NY 10017, USA

**Keywords:** conflict analysis, social fabric, finite element analysis, social vulnerability, resilience modeling, conflict impact, spatial analysis, mathematical modeling

## Abstract

Understanding the societal impacts of armed conflict remains challenging due to limitations in current models, which often apply fixed-radius buffers or composite indices that obscure critical dynamics. These approaches struggle to account for indirect effects, cumulative damage, and context-specific vulnerabilities, especially the question of why similar events produce vastly different outcomes across regions. We introduce a novel computational framework that applies principles from engineering and material science to conflict analysis. Communities are modeled as elastic plates, “social fabrics”, whose physical properties (thickness, elasticity, coupling) are derived from spatial socioeconomic indicators. Conflict events are treated as external forces that deform this fabric, enabling the simulation of how repeated shocks propagate and accumulate. Using a custom Python-based finite element analysis implementation, we demonstrate how heterogeneous data sources can be integrated into a unified, interpretable model. Validation tests confirm theoretical behaviors, while a proof-of-concept application to Nigeria (2018) reveals emergent patterns of spillover, nonlinear accumulation, and context-sensitive impacts. This framework offers a rigorous method to distinguish structural vulnerability from external shocks and provides a tool for understanding how conflict interacts with local conditions, bridging physical modeling and social science to better capture the multifaceted nature of conflict impacts.

## 1. Introduction

Armed conflicts have increased dramatically in recent decades, with the number of major events nearly tripling and battle-related deaths increasing six-fold [1,2]. By 2030, over 60% of the world’s poor are projected to live in fragile and conflict-affected settings [3,4]. Contemporary warfare rarely remains contained within discrete battlefields, creating wide-ranging damage to institutions, economic structures, and community networks far beyond immediate combat zones [5,6].

Sub-Saharan Africa has witnessed a disproportionately high number of conflicts over the past three decades, with adverse effects persisting long after conflicts end [7,8,9]. Approximately 420 million children worldwide lived in conflict-affected areas in 2017, with civilian casualties significantly outnumbering combatant deaths at ratios exceeding five to one [1,9,10]. Studies suggest that conflict-related child mortality may be ten times higher than direct combat estimates when accounting for indirect effects such as health system breakdown and service disruption [9].

The complexity of conflict impacts presents fundamental challenges for analysis. While direct effects include fatalities, displacement, and infrastructure destruction, indirect consequences such as health system breakdown, educational disruption, and economic transformation often overshadow immediate combat mortality [9,10]. Between 1995 and 2015 alone, an estimated 4.9 to 5.5 million deaths of children under five were linked to conflict, corresponding to 6.6 to 7.4% of all deaths in that age group [9]. Critically, these impacts are not confined to “front line” areas, as communities located hundreds of kilometers from active violence still experience psychological distress, nutritional deficiencies, and service breakdowns [11].

Despite increasing availability of conflict datasets such as the Uppsala Conflict Data Program (UCDP) and Armed Conflict Location and Event Data (ACLED), fundamental gaps remain in understanding how violence evolves within particular communities and why similar conflict events lead to vastly different outcomes [10,12]. Large-scale models often assume uniform exposure buffers around conflict incidents, typically 10–50 km, masking localized vulnerabilities and spillover effects [7,11]. These approaches risk significantly underestimating real impacts, especially when indirect and cumulative effects fail to register as “major” events [9,10].

Micro-level surveys provide rich detail about household welfare, child health, and local service capacity, but face safety constraints, reporting biases, and limited spatial coverage in conflict settings [3,13,14]. Insecure regions may be excluded from sampling, and respondents may not disclose sensitive information due to fear of reprisals [13,14]. Multi-indicator frameworks often combine conflict and vulnerability measures into composite indices, but this aggregation limits their ability to model how different factors interact dynamically over space and time [7,15].

A fundamental gap remains: few frameworks preserve a clear mathematical distinction between the “impact” (conflict events) and the “impacted” (community characteristics) [7,10,16]. This distinction is critical for understanding why similar events can produce vastly different outcomes depending on where they occur, and for modeling how repeated shocks accumulate and interact with local vulnerabilities.

Existing approaches to conflict impact assessment highlight this gap. Machine learning frameworks, including the ViEWS consortium, demonstrate the ability to forecast political violence at both national and sub-national levels with ensemble-based methods that integrate multiple thematic models [17]. Advances in automated machine learning (AutoML) further improve accuracy, with applications in African forecasting competitions showing that AutoML models can outperform traditional approaches, particularly when designed to capture spatial and temporal dynamics [18]. A broader review of machine learning in conflict studies emphasizes the decisive role of predictor and conditioning factor selection, noting how governance quality, socioeconomic stressors, and environmental risks systematically condition conflict susceptibility [19]. These insights also highlight potential complementarities, as machine learning techniques could support parameter calibration or data preprocessing within physics-based models.

Operational early warning systems provide another important strand. The ViEWS platform offers publicly available, monthly updated forecasts for multiple forms of political violence across Africa [17]. ACLED has developed complementary operational tools to their datasets, such as the Conflict Exposure methodology, which estimates populations exposed to conflict events through high-resolution population mapping and spatial buffers, updated in near real-time [20]. These systems can provide rapid assessments. However, methodological choices, such as reliance on proximity-based exposure estimates [20] and ensemble modeling thresholds [17], can shape outputs and should be interpreted with their documented trade-offs in mind.

These approaches have made important advances, but they leave the problem of how heterogeneous social conditions mediate the propagation and accumulation of conflict impacts. Our physics-based framework addresses this gap by providing an interpretable, process-oriented alternative. By maintaining explicit distinction between external shocks and system properties, it complements predictive, causal, and operational traditions while offering new insights into the structural mechanisms of resilience and vulnerability.

Inspired by plate theory in continuum mechanics, we conceptualize society as a “social fabric” with material properties derived from local socioeconomic indicators. Violent events are modeled as external forces acting upon a heterogeneous medium, where local material properties determine how impacts propagate, accumulate, and interact. Drawing on established principles from continuum mechanics and finite element analysis (FEA), the framework captures key phenomena identified in conflict literature: spillover effects, cumulative impacts from repeated events, and the critical role of local vulnerabilities in determining impact severity [11,16,21].

This approach addresses three interconnected objectives and offers four key contributions:

Objectives:

- Formulate a conceptual “social fabric” model that translates social characteristics and conflict events into physical analogues within a unified framework
- Demonstrate the framework’s capabilities through a proof-of-concept application showing how compound and protracted conflict scenarios can be simulated
- Establish foundations for future validation and interdisciplinary collaboration by identifying gaps between physics-based modeling and real-world implementation needs

Key contributions:

- A systematic translation system that encodes diverse social indicators into interpretable physical parameters through context-sensitive response functions
- A unified computational model showing how conflict impacts emerge from dynamic interactions between external event stressors and spatially varying social conditions
- A proof-of-concept demonstration using real-world data that captures spillover effects, cumulative damage, and context-sensitive impact patterns
- A conceptual framework treating indirect and cumulative impacts as core emergent features rather than secondary effects, establishing foundations for interdisciplinary research bridging engineering principles with social science insights

The remainder of this paper proceeds as follows: Section 2 outlines our methodology, including the translation framework and implementation details. Section 3 presents validation results and proof-of-concept findings. Section 4 discusses implications, limitations, and future directions. Section 5 presents the conclusions.

## 2. Methods

**Figure 1:**
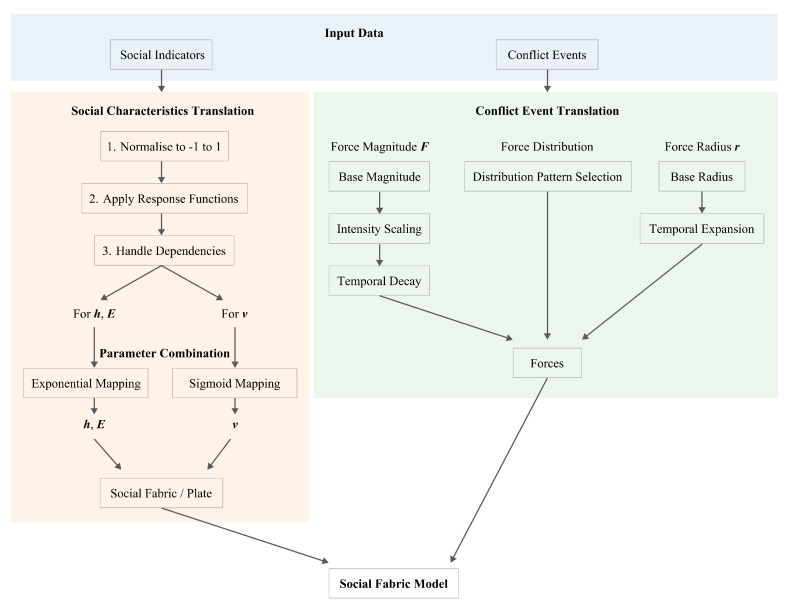
Workflow illustrating the translation of social indicators and conflict events into a computational model of impact.

Figure 1 presents the workflow of our framework. Social indicators and conflict events are translated into a unified computational system that generates spatial displacement fields representing cumulative conflict impact.

### 2.1. Social Fabric Concept

We model conflict-affected regions as a “social fabric” analogous to a thin elastic plate, where communities exhibit characteristic responses to external stresses based on their underlying properties. This physics-based analogy enables systematic encoding of social complexity into quantifiable parameters while maintaining mathematical rigor.

In recent decades, bridging quantitative physical models with the qualitative depth of the social sciences has emerged as a promising route for understanding complex societal phenomena [22,23]. Early work in synergetics underscored that while social systems demand nuanced, qualitative concepts, carefully formulated mathematical models can reveal hidden structures and emergent patterns. Moreover, as [22] suggest, global behaviors in large-scale systems often depend on higher-level “universal” features rather than microscopic details, making it feasible to adapt physical theories for social contexts.

The approach also builds on established vulnerability and resilience theories, particularly the Disaster Resilience of Place (DROP) model [24] and Baseline Resilience Indicators for Communities (BRIC) framework [25], which identify measurable factors contributing to community resilience across multiple domains.

Current approaches to conflict impact assessment face several limitations that motivate this physics-based alternative. Spatial overlay methods use fixed-radius buffers around conflict sites, typically 10–50 km, but miss spillover effects that may extend beyond these boundaries and ignore local context variations [7,9,26]. Multi-indicator frameworks combine conflict and vulnerability measures into composite indices, but this aggregation limits their ability to model dynamic interactions between different factors [15].

Violence exhibits complex spatial and temporal dynamics, including contagion effects where conflict spreads to neighboring areas [5,11,21,26,27] and cumulative impacts where repeated shocks erode community capacity [3,7,9,28]. These phenomena require analytical approaches that can capture both local heterogeneity and system-level interactions.

The framework uses three main material properties, which serve as placeholders for broader social dynamics:

Thickness (*h*) represents a region’s baseline capacity to absorb disruption. In the mechanical model, thickness scales impact absorption nonlinearly. In social terms, this parameter may be interpreted as a region’s capacity to absorb stress without exhibiting large-scale disruption. While abstract, this absorption capacity may be influenced by access to basic services, institutional robustness, and demographic resilience, reflecting concepts from the vulnerability literature [14,24,25,29].

Young’s modulus (*E*) scales linearly with displacement under force, and thus governs how much stress translates into observable change. We use it to represent a region’s resistance to external disruptions. This parameter may be informed by economic strength, access to resources, or social capital. Communities with greater wealth or better resource access may resist disruption more effectively, aligning with findings linking economic resources to reduced vulnerability [16,24,29].

Poisson’s ratio (ν) determines how stress spreads across space, influencing the directional coupling of impact. In social systems, this parameter may loosely correspond to inter-regional connectedness, whether through infrastructure, media, kinship ties, or migration flows. Higher values reflect more coupled regions where stress in one area rapidly propagates to others. Research suggests that such connectivity can amplify both beneficial coordination and harmful cascade effects in crisis contexts [12,16,30].

Each mapping remains interpretive rather than empirical. The parameters do not claim to be infrastructure, wealth, or social ties, but provide a structured way to simulate how those elements, when distributed heterogeneously across space, mediate the propagation of conflict-related impact.

### 2.2. Mathematical Formulation

We apply Kirchhoff–Love plate theory, where the governing differential equation takes this form:

(1) $$D\nabla^{4}w=q$$

where *w* represents vertical displacement, *q* denoted applied forces, and *D* is the bending stiffness given by:

(2) $$D=\frac{Eh^{3}}{12\cdot (1-\nu^{2})}$$

Equation (2) shows how thickness has a cubic relationship with bending stiffness, explaining distinct response regimes. The thickness parameter exhibits three behavioral ranges based on this cubic relationship: below approximately 1000 m, small changes produce large response variations reflecting high vulnerability. The intermediate range (2000–5000 m) shows proportional responses characteristic of moderate resilience, and above 5000 m, additional strengthening yields diminishing returns, representing robust communities with baseline resilience [10,11,14].

Equation (1) ensures that displacement patterns emerge from the interaction between material properties (encoding social characteristics) and applied forces (representing conflict events). The bi-harmonic operator $\nabla^{4}$ captures how impacts propagate through the continuous medium, enabling modeling of spillover effects and cumulative damage [31,32].

The linear elastic approximation provides several advantages as a starting point: it enables superposition of multiple events, creates symmetric scaling between enhancement and reduction effects, and allows clear interpretation of parameter influences. While real communities may exhibit plastic deformation or failure under extreme stress, the linear approach offers mathematical tractability while capturing key phenomena identified in conflict literature [22,31].

We implement the finite element method (FEM) using custom Python code that handles geographic coordinate systems, spatially varying material properties, and flexible boundary conditions [33]. Comprehensive behavioral analysis of uniform and heterogeneous plate properties, boundary condition effects, and force interactions is presented in the extended, pre-print thesis version [34]. A minimum 300 km buffer zone around regions of interest ensures boundary effects do not influence results within the analysis area.

### 2.3. Translation Framework: Social Indicators to Material Properties

Social indicators are systematically translated into material properties through a three-step process: normalization, response function application, and parameter combination. This translation framework represents a key methodological contribution, enabling diverse socioeconomic data to be encoded into physical parameters.

#### 2.3.1. Normalization and Response Functions

Each indicator is first normalized to ensure consistent scaling across different data types using:

(3) $$x_{normalized}=\frac{x_{actual}-x_{mid}}{x_{max}-x_{mid}}$$

where $x_{mid}$ represents neutral conditions, typically the median or a theoretically meaningful baseline. This normalization maps indicators to the range [−1, 1], where negative values represent vulnerability effects and positive values represent resilience effects.

Each normalized value is then passed through a response function that reflects how social indicators affect a given physical property. We define five core types based on different behavioral patterns:

Linear response creates directly proportional modifications to material properties. For instance, ref. [25] mention the number of public schools per 10,000 as an indicator of school restoration potential. Each incremental change in the number of schools would create a proportional change in community resilience. The slopes $m_{v}$ and $m_{r}$ control how strongly changes negatively (vulnerability) or positively (resilience) modify the plate property:

(4) $$f\left(x\right)=\left\{\begin{array}{cc} -1\cdot m_{v}x & \;\mathrm{if}\;x<0\;\left(\mathrm{vulnerability}\right),\mathrm{with}\;m_{v}>0\\ +1\cdot m_{r}x & \;\mathrm{if}\;x\geq 0\;\left(\mathrm{resilience}\right),\mathrm{with}\;m_{r}>0 \end{array}\right.$$

Logarithmic response creates strong initial effects that diminish as the indicator value increases in magnitude. This suits institutional indicators like disaster preparedness knowledge [25]. Initial implementations of disaster preparedness training might create significant improvements in baseline resilience, but additional expansions yield progressively smaller enhancements. The parameters $\alpha_{v}$ and $\alpha_{r}$ control how quickly diminishing returns set in:

(5) $$f\left(x\right)=\left\{\begin{array}{cc} -1\cdot \ln(1+\alpha_{v}|x|) & \;\mathrm{if}\;x<0\;\left(\mathrm{vulnerability}\right),\mathrm{with}\;\alpha_{v}>0\\ +1\cdot \ln(1+\alpha_{r}|x|) & \;\mathrm{if}\;x\geq 0\;\left(\mathrm{resilience}\right),\mathrm{with}\;\alpha_{r}>0 \end{array}\right.$$

Exponential response captures indicators where effects intensify progressively as values become more extreme. This pattern is relevant for indicators with minimal impact near-neutral conditions but rapidly growing effects as they deviate further. For example, jurisdictional coordination complexity, where increasing numbers of governments create rapidly accelerating coordination breakdown potential [25]. The parameters $\beta_{v}$ and $\beta_{r}$ control acceleration rate:

(6) $$f\left(x\right)=\left\{\begin{array}{cc} -1\cdot (e^{\beta_{v}\left|x\right|}-1) & \;\mathrm{if}\;x<0\;\left(\mathrm{vulnerability}\right),\mathrm{with}\;\beta_{v}>0\\ +1\cdot (e^{\beta_{r}\left|x\right|}-1) & \;\mathrm{if}\;x\geq 0\;\left(\mathrm{resilience}\right),\mathrm{with}\;\beta_{r}>0 \end{array}\right.$$

Power law response provides flexible curvature between logarithmic and exponential behaviors. With γ<1, it produces stronger initial effects that level off dramatically, suitable for indicators where small improvements from poor conditions create substantial gains but additional improvements yield diminishing returns. With γ>1, it creates late-state acceleration for indicators requiring considerable levels before significant effects occur:

(7) $$f\left(x\right)=\left\{\begin{array}{cc} -1\cdot (|x{|}^{\gamma_{v}}) & \;\mathrm{if}\;x<0\;\left(\mathrm{vulnerability}\right),\mathrm{with}\;\gamma_{v}>0\\ +1\cdot (|x{|}^{\gamma_{r}}) & \;\mathrm{if}\;x\geq 0\;\left(\mathrm{resilience}\right),\mathrm{with}\;\gamma_{r}>0 \end{array}\right.$$

Sigmoid response represents threshold behavior with stable states at both extremes and concentrated transition zones. This creates S-shaped curves where initial changes produce minimal effects, followed by rapid transition, before stabilizing. This is relevant for community resilience collapse or overload phenomena [35]. The transition point $x_{0}$ determines where change occurs, while steepness parameter *k* controls transition abruptness:

(8) $$f\left(x\right)=\left\{\begin{array}{cc} -1\cdot (\frac{1}{1+e^{-(k_{v}\left(|x\right|-x_{0_{v}}))}}) & \;\mathrm{if}\;x<0\;\left(\mathrm{vulnerability}\right),\mathrm{with}\;k_{v}>0\\ +1\cdot (\frac{1}{1+e^{-(k_{r}\left(|x\right|-x_{0_{r}}))}})) & \;\mathrm{if}\;x\geq 0\;\left(\mathrm{resilience}\right),\mathrm{with}\;k_{r}>0 \end{array}\right.$$

The choice of a response function should reflect empirical knowledge or theoretical expectations about how different social characteristics influence community behavior [25,36].

#### 2.3.2. Dependencies and Parameter Combination

Social systems exhibit critical interdependencies that must be considered in the translation process. For example, healthcare infrastructure effectiveness may depend on accessibility, as even well-equipped facilities provide limited benefit if they are unreachable during crises [37]. Similarly, water infrastructure systems may completely fail without electrical power for pumps, regardless of the water system’s own robustness [38].

The framework addresses these dependencies through two main mechanisms:

Threshold dependencies capture situations where one indicator’s effectiveness requires another to meet minimum levels. This is modeled as:

(9) $$f_{effective_{B}}\left(x_{A},x_{B}\right)=\left\{\begin{array}{cc} base_{B}\cdot f_{B}\left(x_{B}\right) & \mathrm{if}\;f_{A}\left(x_{A}\right)<threshold\\ f_{B}\left(x_{B}\right) & \mathrm{if}\;f_{A}\left(x_{A}\right)\geq threshold \end{array}\right.$$

where indicator *B* depends on indicator *A*. When the prerequisite indicator *A* falls below the threshold, *B*’s influence reduces to a baseline fraction of its full effect.

Amplifying/dampening dependencies model situations where one indicator modifies another’s impact through multiplicative effects:

(10) $$f_{compound_{B}}\left(x_{A},x_{B}\right)=f_{B}\left(x_{B}\right)\cdot \left(1+f_{A}\left(x_{A}\right)\cdot c_{AB}\right)$$

where $c_{AB}$ represents the strength of the interaction effect. Positive values create amplifying effects where strong institutions enhance resource deployment, while negative values create dampening effects where weak governance undermines available resources.

After accounting for dependencies, multiple indicators affecting the same parameter are combined mathematically. For thickness and Young’s modulus, we use exponential mapping:

(11) $$p_{combined}=p_{base}\cdot \exp\left(\sum_{i=1}^{n}w_{i}f_{i}\left(x_{i}\right)\right),\;\mathrm{with}\;\sum_{i=1}^{n}w_{i}=1$$

where $p_{base}$ represents a neutral baseline value, $w_{i}$ are importance weights that sum to 1, and $f_{i}\left(x_{i}\right)$ are the response function outputs for each indicator. This exponential approach ensures parameters remain strictly positive (as required by physics), resilience and vulnerability effects scale symmetrically, and the baseline represents truly neutral conditions.

For Poisson’s ratio, the physical constraint 0<ν<0.5 requires a different approach using sigmoid-based mapping [39]:

(12) $$\nu_{combined}=\frac{0.5}{1+\exp(k\cdot \sum_{i=1}^{n}f_{i}\left(x_{i}\right)\cdot w_{i})},\;\mathrm{with}\;\sum_{i=1}^{n}w_{i}=1$$

where *k* controls the sensitivity of the mapping. This formulation automatically maintains bounds while allowing vulnerability indicators (negative $f_{i}$) to increase Poisson’s ratio (stronger coupling) and resilience indicators (positive $f_{i}$) to decrease it (more localized effects).

### 2.4. Translation Framework: Conflict Events to Forces

Conflict events are translated into forces through a comprehensive mapping that captures their varied characteristics across three key dimensions: magnitude, distribution pattern, and spatial radius. This translation preserves the temporal evolution of conflicts while encoding their diverse impacts into the physical force framework.

#### 2.4.1. Force Magnitude

The total force magnitude for event *e* at time *t* combines base event characteristics with intensity scaling and temporal decay:

(13) $$F_{e}\left(t\right)=F_{base}\left(e\right)\cdot I\left(e\right)\cdot T\left(\Delta t\right),\;\mathrm{with}\;\Delta t=t-t_{e}$$

where $\Delta t=t-t_{e}$ represents their time elapsed since the event occurred.

Base event magnitude $F_{base}\left(e\right)$ establishes a hierarchy of impact potential across different conflict types. The specific values assigned depend on the context and available empirical evidence about relative event impacts. Domain experts can calibrate these magnitudes based on local knowledge, historical data, or theoretical considerations about relative destructive capacity and societal impact.

For protest, democratic context significantly influences interpretation, following established methodologies by [15]. The base magnitude is adjusted by $\left(1-V_{dem}\right)$ where $V_{dem}$ is the V-Dem liberal democracy index, ensuring protests carry appropriate weight in different political contexts [40].

Event intensity scaling incorporates fatality data and targeting characteristics:

(14) $$I\left(e\right)=S\left(n_{f}\right)\cdot \left(1+\gamma_{c}\cdot C_{t}\right)$$

The fatality scaling function $S\left(n_{f}\right)=\alpha_{c}\left(1+n_{f}\right)+\left(1-\alpha_{c}\right)\left(1+\log\left(1+n_{f}\right)\right)$ balances linear and logarithmic scaling based on event types, where $\alpha_{c}$ determines the relative weight of each approach. Linear scaling assumes proportional impact increases with additional fatalities, while logarithmic scaling captures diminishing marginal effects where initial deaths create substantial societal shock but additional casualties produce smaller incremental impacts.

The civilian targeting multiplier $\left(1+\gamma_{c}\cdot C_{t}\right)$ accounts for the documented finding that civilian-targeted violence often produces indirect casualties exceeding direct fatalities by over 75% [10,27]. Setting the parameter to $\gamma_{c}=0.75$ would reflect this observation, while $C_{t}\in 0,1$ indicates whether civilians were primary targets or not.

Temporal decay models how event impacts diminish over time while maintaining persistent influence:

(15) $$T\left(\Delta t\right)=\left\{\begin{array}{cc} \exp(-\lambda \cdot \Delta t) & \mathrm{if}\;\Delta t<\frac{\ln(100)}{\lambda }\\ 0 \end{array}\right.$$

The exponential decay function ensures events maintain diminishing but meaningful influence over time appropriate to their characteristics, reflecting that conflict events can persist well beyond their immediate aftermath [3,15]. The decay rate λ can be calibrated based on empirical evidence about impact persistence, theoretical considerations about different event types, or expert knowledge about local contexts. The cutoff mechanism ensures computational efficiency by removing events once their influence drops to 1% of their original magnitude.

#### 2.4.2. Force Distribution and Radius

Four patterns capture the spectrum from highly localized to broadly distributed effects in this framework:

Point forces apply the entire magnitude to a single location, appropriate for highly targeted violence affecting specific individuals or very small groups, such as targeted assassinations or abductions.

Gaussian distributions create concentrated impacts with rapid distance decay, possibly suitable for precision military strikes (air/drone strikes, targeted bombings) where effects concentrate strongly at recorded coordinates but acknowledge some spatial uncertainty and immediate surrounding impacts.

Linear distributions produce gradual decline from the epicenter, appropriate for events with clear focal points but substantial direct effects in surrounding areas, such as battles where fighting intensity decreases with distance from the main location.

Constant distributions spread forces uniformly across the application area, suitable for events creating widespread but relatively uniform effects, such as protests that may move through urban areas or riots affecting entire districts.

The spatial radius of each event evolves over time to simulate the delayed diffusion of conflict impact. This dynamic radius $r_{e}\left(t\right)$ is defined as:

(16) $$r_{e}\left(t\right)=r_{base}\left(e\right)\cdot E\left(\Delta t\right),\;\mathrm{with}\;\Delta t=t-t_{e}$$

Here, $r_{base}\left(e\right)$ is the initial radius assigned to each event type, and E(Δt) is a temporal expansion function capturing indirect and delayed effects such as news diffusion, psychological trauma, and social contagion [11].

The expansion function is defined as:

(17) $$E\left(\Delta t\right)=1+\beta_{c}\cdot \left(1-\exp\left(-\mu \cdot \Delta t\right)\right)$$

This function is governed by two parameters: the maximum relative expansion factor $\beta_{c}$, which determines how much the radius can grow beyond its initial extent, and the expansion rate μ, which controls how quickly this growth occurs. Initially, E(Δt)≈1, but as time progresses, it asymptotically approaches $1+\beta_{c}$.

This function enables flexible modeling of both rapid-shock and gradual-diffusion phenomena. Importantly, these radii define only the zones of direct event impact, not the total affected areas. Broader spillover effects and indirect impacts emerge naturally from the interaction between these localized forces and the spatially varying social fabric properties.

### 2.5. Implementation and Mechanical Validation

The framework generates finite element meshes with 10 km resolution, assigns spatially varying material properties from social indicator mappings using Equations (3)–(12), applies force representation of conflict events using Equations (13)–(16), and solves Equation (1) for displacement fields representing cumulative impact. The implementation is built entirely on widely adopted Python libraries (NumPy 2.1.3 [41], SciPy 1.14.1 [42], Matplotlib 3.9.2 [43], GeoPandas 1.0.1 [44], Rasterio 1.4.2, Pandas 2.2.3 [45], Plotly 6.2.0 [46], Contextily 1.6.2) without requiring additional GIS or specialized software, ensuring maximum accessibility and reproducibility. Its reliability is verified through comprehensive testing including element mathematics, eigenvalue analysis, mesh connectivity, and boundary condition validation available in the open-source code [33], with systematic behavioral analysis detailed in the extended pre-print thesis [34]. Validation testing confirmed expected physical behaviors: thickness effects follow cubic scaling (as shown in Equation (2)), Young’s modulus shows linear displacement relationships, and multiple forces demonstrate appropriate superposition behaviors consistent with the mathematical formulation.

## 3. Results

### 3.1. Framework Validation

We validated the framework using a plate approximating Nigeria’s dimensions (1620 km × 1370 km) with 10 km mesh resolution and 350 km buffer zones. Testing focused on verifying the expected physical behavior of the computational model, ensuring it adheres to theoretical properties of linear elasticity and finite element implementation.

**Figure 2:**
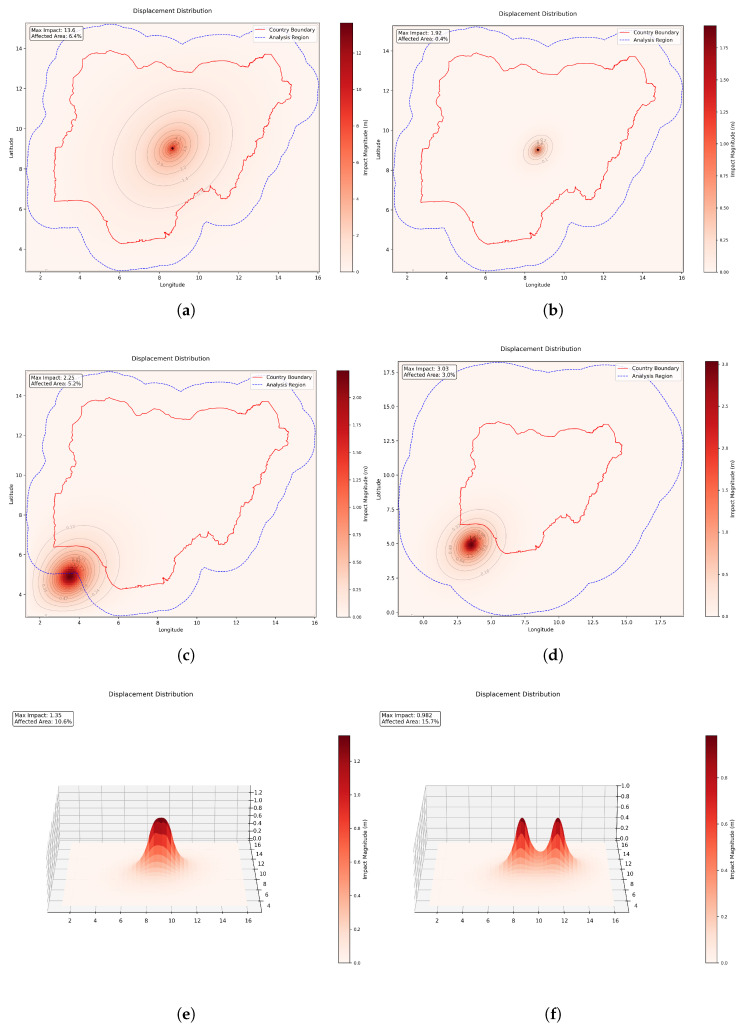
Framework validation results demonstrating expected physical behaviors. (**a**,**b**) Thickness effects comparing a thin plate (**a**) (1000 m thickness) showing broad impact distribution with thick plate (**b**) (5000 m thickness) demonstrating localized response. (**c**,**d**) Boundary condition independence comparing 150 km buffer (**c**) with 500 km buffer (**d**), confirming that adequate buffer zones eliminate edge effects and ensure reliable results within the region of interest. (**e**,**f**) Force interaction patterns showing displacement fields for two equal forces a 1° separation (**e**) creating overlapping effects and 3° separation (**f**) producing more independent responses.

Figure 2 presents key validation results. Thickness behavior followed the cubic scaling described by Equation (2), as shown by the differences in the displacement fields between a thin plate (1000 m) and a thick plate (5000 m) (Figure 2a,b). Young’s modulus demonstrated perfect linear inverse scaling across six orders of magnitude while maintaining constant spatial distribution patterns, validating its role as a pure scaling factor. Poisson’s ratio, constrained to the range [0, 0.5), showed moderate but consistent effects on both displacement magnitude and spatial localization.

Boundary condition testing established that buffer zones exceeding 300 km effectively eliminate computational edge effects. At 150 km buffer (Figure 2c), maximum displacement errors near boundaries reached 2.4%, reducing to 0.4% at 500 km buffer (Figure 2d), confirming the reliability of results within regions of interest.

Multiple force interactions confirmed proper superposition behavior essential for modeling overlapping conflicts. Two equal forces at 1° separation produced 43% higher maximum displacement than single-force scenarios (Figure 2e), while forces at 3° separation remained largely independent (Figure 2f). Force distribution patterns significantly influenced impact characteristics: Gaussian distributions created the highest peak displacement (4.74 m) with smallest affected area (2.77%), while constant distributions produced broader impact zones (7.75% affected area) with lower peaks (1.89 m).

Finally, displacement was confirmed as a more suitable impact metric than von Mises stress. Displacement fields showed consistent behavior with highest values at force centers, smooth gradients across space, and intuitive interaction patterns. Von Mises stress produced counter-intuitive, but physically expected, results in areas of overlap and near event centers, making displacement the preferred measure for social impact modeling.

In this context, displacement refers specifically to the physical deformation of the modeled plate structure measured in meters, representing cumulative conflict impact intensity, which differs from the humanitarian use of “displacement” to describe forced human migration. This physical displacement metric demonstrates superior behavior in capturing spillover effects and cumulative damage compared to alternative stress measures, while the semantic distinction from population displacement ensures clarity when communicating results to interdisciplinary audiences.

These findings establish that the computational implementation behaves in accordance with theoretical expectations, confirming its suitability for spatial conflict modeling under the assumptions of linear elasticity.

### 3.2. Proof-of-Concept Application: Nigeria 2018

We demonstrated the framework’s practical application using Nigeria as a test case, combining ACLED conflict data with spatially distributed social indicators to model cumulative conflict impact throughout 2018 [47]. This proof-of-concept illustrates operational feasibility and produces plausible outputs, though parameter choices are assumption-based rather than empirically validated.

#### 3.2.1. Social Fabric Construction

To construct the social fabric, we translated nine spatially distributed social indicators into three core material parameters: thickness (*h*), Young’s modulus (*E*), and Poisson’s ratio (ν). These parameters do not carry fixed social meanings but serve as interpretable placeholders for absorptive capacity, resistance, and propagation dynamics, respectively. The full mapping specifications, including data sources, transformation functions, and weights, are provided in Appendix A, Table A1.

Indicator selection followed established vulnerability and resilience frameworks while prioritizing spatial coverage and temporal alignment.

Thickness was shaped by a composite of critical infrastructure density [48], healthcare accessibility [37,48], environmental stress [49], and demographic dependency [50]. To demonstrate the dependency framework, healthcare infrastructure effectiveness was modeled as dependent on accessibility using a threshold dependency: when walking time to healthcare facilities exceeded 30 min, the infrastructure’s resilience effect was reduced to 40% of its potential impact, reflecting how even well-equipped facilities provide limited benefit if unreachable.

**Figure 3:**
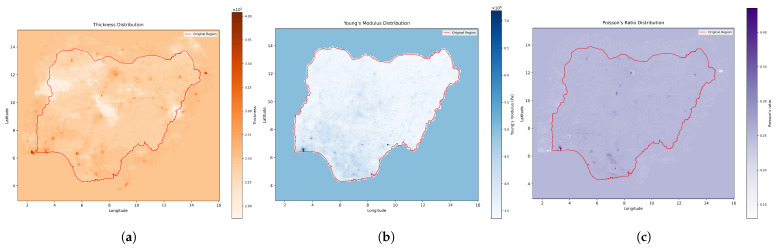
Final physical parameter distributions prepared for FEA implementation: (**a**) Spatial distribution of the combined thickness *h*, derived from the weighted effects of various indicators. Values range from approximately 2,000 m (lower resilience) to 4,000 m (higher resilience); (**b**) Spatial distribution of Young’s modulus *E*, derived from the weighted effects of various indicators. Values range from approximately $3.5\times 10^{9}$ Pa (higher sensitivity) to $7.0\times 10^{9}$ Pa (lower sensitivity); (**c**) Spatial distribution of Poisson’s ratio ν, derived from the weighted effects of various indicators. Values range from approximately 0.15 (more localized impact propagation) to 0.4 (stronger impact coupling). The mesh grid overlay illustrates the 10 km spatial discretizations used for the finite element implementation.

Regions with robust service coverage and lower environmental pressure, such as parts of the southwest, exhibited higher thickness values, reflecting stronger absorptive capacity (Figure 3a). By contrast, areas in the northwest showed lower thickness, capturing the effects of compounding vulnerabilities where limited infrastructure intersects with harsh ecological conditions and higher dependent populations.

Young’s modulus was mapped using gridded economic indicators, including GDP per capita [51], poverty rates [52], and childhood deprivation metrics [53]. These were combined to reflect economic resilience and institutional strength. Urbanized regions such as Lagos and Abuja displayed higher stiffness values, suggesting greater resistance to deformation under stress (Figure 3b). Rural and economically marginalized zones, meanwhile, showed significantly lower values, indicating greater susceptibility to disruption even under moderate force exposure.

Poisson’s ratio was derived from population density and road network data [54,55], which together reflect both social and physical connectivity. Densely populated and well-connected areas demonstrated higher lateral coupling, allowing stress to spread more widely (Figure 3c). In contrast, low-density and poorly connected areas showed more localized responses. The resulting ν values ranged from 0.15 in isolated regions to 0.4 in metropolitan centers.

#### 3.2.2. Conflict Event Translation

We translated conflict events into spatially and temporally evolving force fields by applying the framework to 1190 geolocated events recorded in Nigeria during 2018, sourced from the ACLED database [47,56]. Events lacking precise coordinates (i.e., with lower location precision codes) or classified as strategic developments were excluded, ensuring fidelity in spatial modeling [56]. The event sample was diverse, comprising protests (32.2%), violence against civilians (26.6%), battles (21.5%), riots (15.8%), and explosions or remote violence (3.9%). Detailed classifications are provided in Appendix B, Table A2 and Table A3.

Force magnitudes were assigned based on estimated relative impact potential, anchored to a reference value of $1\times 10^{9}$ N for explosions and remote violence, reflecting their concentrated destructive power [57]. Other event types were assigned scaled magnitudes: battles at 80%, violence against civilians at 60%, riots at 30%, and protests at 10%, with further adjustment for democratic context using the V-Dem liberal democracy index [40]. These ratios were informed by literature on conflict lethality and assumed impact hierarchies but are presented as provisional, subject to future empirical calibration [57].

Intensity scaling combined fatality counts and civilian targeting using Equation (14). Fatalities were scaled with a hybrid linear–logarithmic function to capture both proportional and diminishing-return effects. Targeting of civilians was incorporated using the multiplier $\left(1+\gamma_{c}\cdot C_{t}\right)$, with $\gamma_{c}=0.75$ representing documented indirect mortality rates [10]. This formulation ensured that both severity and target type influence total impact.

Temporal decay followed the exponential formulation described in Equation (15), with decay rates assigned per event type. Explosions and battles were assumed to have longer-lasting effects (approximately two years), reflecting infrastructure damage and prolonged displacement. Violence against civilians was modeled with a one-year decay window, while riots and protests decayed over approximately six months. These durations are consistent with theoretical assumptions but remain open to empirical validation.

Events were assigned one of four spatial distribution types to reflect their diffusion behavior based on the physical characteristics of different conflict types [57]. Point forces apply the entire magnitude to a single location, appropriate for highly targeted violence affecting specific individuals (e.g., assassinations, abductions). Gaussian distributions create concentrated impacts with rapid distance decay, suitable for precision military strikes (e.g., airstrikes, targeted bombings) where effects concentrate strongly at recorded coordinates but acknowledge spatial uncertainty. Linear distributions produce gradual decline from the epicenter, appropriate for events with clear focal points but substantial direct effects in surrounding areas (e.g., battles where fighting intensity decreases with distance). Constant distributions spread forces uniformly across the application area, suitable for events creating widespread but relatively uniform effects (e.g., protests that may move through urban areas, riots affecting entire districts).

Base spatial radii ranged from 5 to 25 km depending on event type, with protests/riots assigned smaller radii (5 km) reflecting localized public disruption, explosions/remote violence assigned moderate radii (10 km) based on immediate weapon effects, and battles assigned larger radii (25 km) accounting for combat operations across broader areas. Radii evolved over time using the temporal expansion function defined in Equation (17), capturing delayed effects such as news diffusion and psychological trauma [21].

Importantly, these radii define only the zones of direct event impact, not the total affected areas. Broader spillover effects and indirect impacts emerge naturally from the interaction between these localized forces and the spatially varying social fabric properties, distinguishing this approach from traditional buffer-based methods that assume uniform impact within fixed radii.

This structured mapping process ensured that each event was represented with temporally evolving force characteristics, spatially differentiated by event type, and responsive to both social context and intensity. The complete conflict event mapping parameters are summarized in Table A4, Table A5 and Table A6 in Appendix C, including force magnitudes (Table A4), distribution patterns (Table A5), and radius parameters (Table A6).

**Figure 4:**
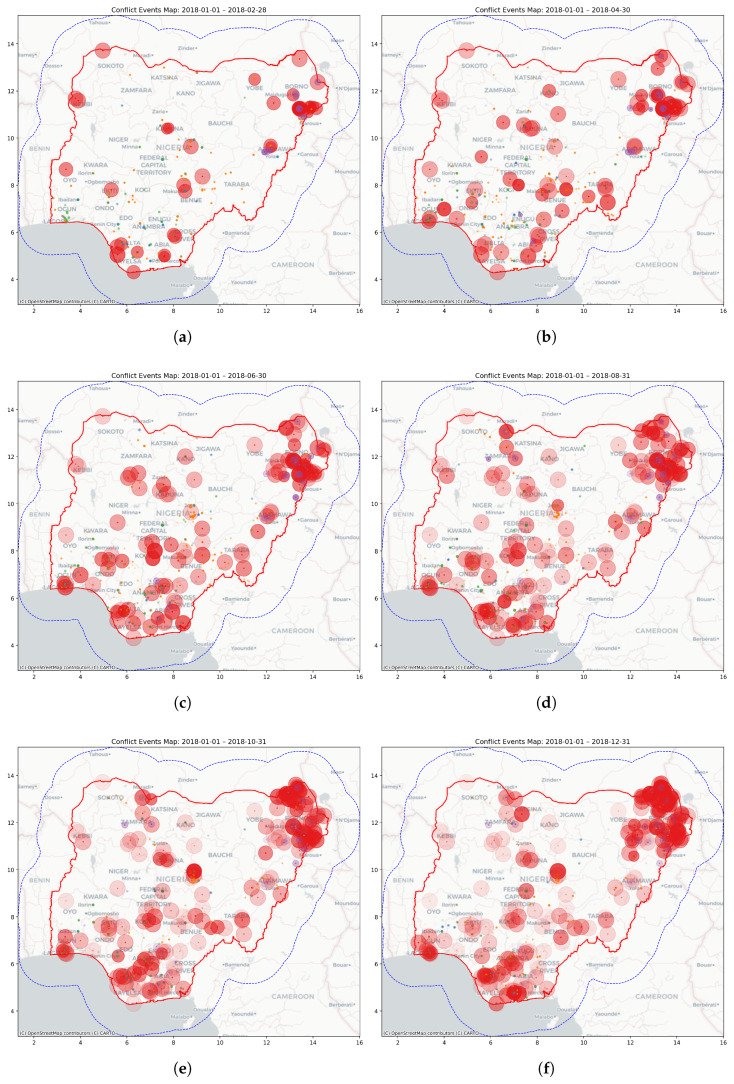
Conflict events for Nigeria in 2018, displayed in six cumulative snapshots labeled (**a**–**f**) in bimonthly increments. The circles are color-coded by event type: battles in red, explosions/remote violence in purple, violence against civilians in orange, riots in blue, and protests in green. Circle transparency indicates temporal decay, with older events appearing more translucent. The red boundary marks the national border, while the dotted blue boundary denotes the buffer region.

Figure 4 shows the temporal evolution of conflict events throughout 2018, demonstrating how force magnitude and spatial extent change over time according to the mapping parameters in Table A4 and Table A6.

#### 3.2.3. Cumulative Impact Results

**Figure 5:**
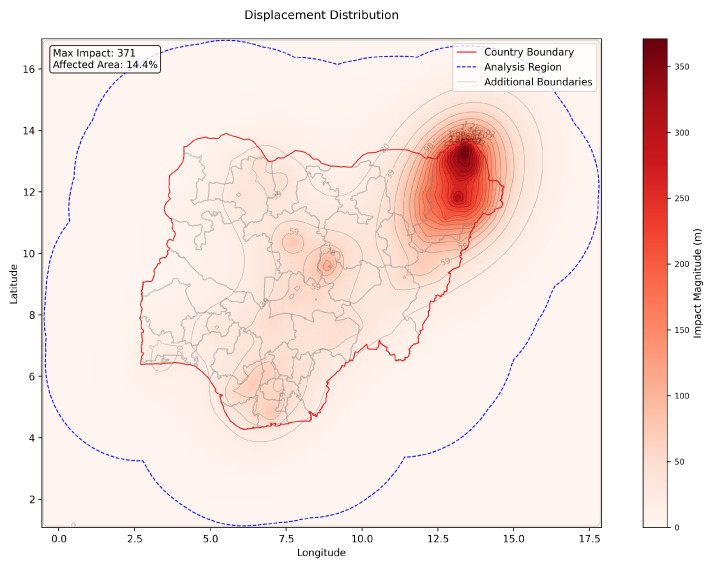
Two-dimensional contour displacement visualization showcasing the proof-of-concept results for conflict events in Nigeria in 2018.

**Figure 6:**
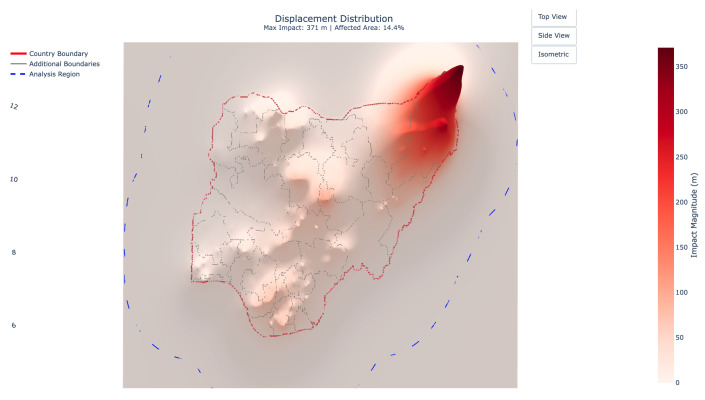
Top view of the 3D displacement visualization showcasing the proof-of-concept results for conflict events in Nigeria in 2018.

The final displacement field revealed a maximum displacement of 371 m, with approximately 14.4% of the total area experiencing significant impact (defined as exceeding 10% of the maximum displacement). Figure 5 and Figure 6 present these results in both two- and three-dimensional visualizations, revealing several notable patterns.

Southwestern regions, characterized by stronger underlying social fabric properties (as shown in Figure 3), exhibited minimal displacement, even in the presence of conflict events. In contrast, the northeast, affected by overlapping high-intensity events and underlying vulnerabilities, displayed severe displacement peaks. These results underscore the model’s capacity to show that impact outcomes depend not merely on event characteristics, but critically on local social conditions.

The framework captured nonlinear accumulation in areas exposed to repeated or clustered events. In high-resilience regions, individual events produced contained, localized effects. In contrast, similar events in vulnerable zones generated broader displacement fields, with the compound effects magnifying stress across space. This behavior aligns with observed cumulative damage dynamics in conflict literature and demonstrates the model’s ability to represent them without post hoc tuning.

Impact propagation extended far beyond event epicenters, with displacement gradients shaped by local material properties rather than uniform buffers. Well-connected regions showed broader but more diffuse stress fields, while isolated communities exhibited sharp displacement peaks. These emergent spillover patterns arise directly from the physics-based interactions between external forces and spatial heterogeneity, without requiring imposed spatial correlation assumptions.

#### 3.2.4. Emergent Behaviors and Key Findings

The results reveal several critical dynamics that are typically missed by traditional conflict analysis approaches. Most notably, the framework captures how identical events produce divergent outcomes depending on local conditions. For example, a protest in Lagos yields a vastly different displacement profile than one in rural Borno State, due to differences in social fabric properties (see Figure 3). This illustrates the model’s ability to represent context-sensitive impacts based on structural vulnerability and resilience.

The temporal formulation (Equation (15)) enables the model to reflect how past events continue to exert pressure on communities even as new ones emerge. This temporal accumulation addresses a key limitation of snapshot-based methods that fail to capture the compounding nature of conflict impacts over time [3,15]. The result is a dynamic model capable of simulating both acute shocks and long-term stress accumulation.

Spatial propagation patterns emerge from the interaction between event forces and the heterogeneous social fabric, following networks of connectivity and vulnerability rather than simple geographic proximity. This leads to non-uniform impact footprints that align more closely with observed conflict spillover patterns and help explain why some areas suffer disproportionately despite being equidistant from violent events [9].

With appropriate calibration, the framework could support early warning applications by identifying regions nearing critical displacement thresholds. This would allow practitioners to monitor cumulative stress and anticipate tipping points where additional shocks might trigger systemic breakdowns.

Overall, the findings validate the framework’s ability to encode complex social conditions into interpretable physical parameters while producing emergent behaviors consistent with conflict research. While this proof-of-concept establishes methodological feasibility, further empirical validation will be necessary to confirm predictive accuracy and operational relevance.

## 4. Discussion

### 4.1. Methodological Contributions

This study introduces a novel application of continuum mechanics and finite element analysis to model the social impact of violent conflict. By conceptualizing society as a heterogeneous elastic plate, the framework translates conflict events into force fields and social indicators into material properties, enabling systematic analysis of how stress propagates through vulnerable systems.

A core contribution lies in the mathematical separation of “impact” and “impacted”. Unlike composite indices that conflate conflict intensity and community vulnerability [15], this framework preserves distinct roles for conflict events (external forces) and local conditions (material parameters). This distinction enables causal modeling, forward simulation, and scenario analysis under varying assumptions, providing a basis for testing the effects of hypothetical interventions or structural changes.

Spatial heterogeneity is modeled explicitly, contrasting with fixed-radius approaches that assume uniform propagation [7,26]. The framework allows for localized variation in both intensity and extent of impacts, demonstrating how identical events produce divergent outcomes depending on local conditions [10,16]. Unlike regression-based methods that treat social indicators as independent variables, our model transforms them into system properties governing response dynamics [11,26].

The physical analogy bridges discrete events and continuous social processes. Rather than aggregating incidents over time or imposing ad hoc assumptions about cumulative effects, the model applies the superposition principle inherent in linear elasticity. The result is a scalable, generalizable framework for simulating emergent behaviors from localized conflict events across spatial and temporal dimensions.

### 4.2. System Behavior and Validation

Model validation confirmed that the computational implementation exhibits expected physical behavior: nonlinear sensitivity to material differences, spatially structured propagation, and aggregation of local stresses into regional patterns. Superposition tests and buffer condition analysis established that the model behaves in accordance with elasticity theory, while displacement proved to be a more interpretable and stable outcome metric than alternative stress measures.

The model captures phenomena widely reported in conflict literature, including spillover, cumulative stress, and delayed effects, not through post hoc assumptions or parameter tuning, but as emergent features of the underlying equations [11,16,21]. While this reinforces the theoretical soundness of the framework, empirical validation remains essential for assessing real-world accuracy and generalization.

### 4.3. Limitations and Future Research

Despite its novelty and rigor, the framework has several limitations that warrant further development. First, it assumes static material properties, ignoring how prolonged violence may degrade infrastructure, alter demographics, or trigger institutional collapse [3]. Extending the model to include dynamic or plastic behavior would allow for capturing irreversible system transformations, tipping points, and changes in community resilience over time.

Second, parameter values used in this proof-of-concept are assumption-based, serving to demonstrate framework capabilities rather than provide operational predictions. Key assumptions include event magnitude hierarchies (with explosions/remote violence as reference events and other types scaled proportionally), temporal decay rates derived from theoretical considerations about impact persistence, response function shapes reflecting presumed relationships between social indicators and system properties, and force distribution patterns assigned based on event type characteristics. While informed by literature and grounded in plausible logic, they lack systematic empirical calibration, focusing only on enabling demonstration of emergent behaviors. Future work should therefore focus on case studies in data-rich conflict settings, where observed outcomes can be used to validate model behavior and refine parameter estimates.

Data limitations remain a further fundamental constraint. Conflict event data are known to suffer from spatial and reporting biases [56], while socioeconomic indicators often lack temporal alignment or sufficient resolution. These limitations highlight the need for systematic sensitivity analysis and uncertainty quantification to ensure outputs are interpreted appropriately given the quality of the underlying data.

The absence of empirical validation against observed humanitarian outcomes represents the most significant limitation. The displacement metric produces relative measures that may support comparative assessments across regions but cannot be directly mapped to specific humanitarian indicators, like mortality, migration, or infrastructure damage without systematic calibration. Establishing quantitative relationships between model outputs and observed outcomes will require a structured, multi-stage validation effort across multiple conflict contexts with access to ground-truth data. At present, the framework demonstrates its capacity to reproduce theoretical behaviors, such as spillover effects, cumulative damage, and context-sensitive responses, but these emergent patterns require empirical grounding before the framework can be used in operational decision-making. In its current form, it should be regarded as a methodological proof-of-concept rather than a predictive tool.

A methodological road map for empirical validation can be outlined to guide future research. An initial stage would involve systematic correlation analysis between model displacement fields and humanitarian indicators such as mortality rates (from conflict event databases), forced displacement figures (e.g., UNHCR, IOM), infrastructure damage assessments (e.g., from satellite imagery), and service disruption metrics (from humanitarian situation reports). Demonstrating that higher simulated model displacement magnitudes consistently correspond with more severe humanitarian outcomes would establish whether the physical metric captures meaningful dimensions of vulnerability rather than remaining a purely mathematical construct.

Building on correlation analysis, comparative case studies across multiple conflict settings are required to test the adoption and transferability to different contexts. Applications spanning urban and rural environments, different conflict types, and varied social fabric mappings would help reveal both universal mechanisms and context-specific requirements. This process would require partnerships with humanitarian organizations for ground-truth data, collaboration with local research institutions for contextual validation, and engagement with national statistical offices for reliable outcomes measures. Multi-context evaluation would clarify whether the model reflects broadly applicable structural dynamics or requires localized parameter adaption.

The most rigorous stage of validation would involve predictive performance assessment. Training the framework on early conflict periods and comparing its outputs against subsequently observed outcomes would test whether the model can anticipate humanitarian consequences rather than merely reproduce past patterns. Benchmarking predictive accuracy against established conflict early warning systems would further situate the contribution of physics-based modeling, highlighting whether it provides distinct advantages in foresight while acknowledging its limitations in rapidly evolving scenarios.

Successful validation will depend on access to sub-national indicators at consistent spatial resolution, temporal alignment between conflict events and humanitarian outcomes, and social datasets with documented uncertainty estimates. Until such calibration is completed, the framework should be considered exploratory: a novel theoretical and methodological foundation awaiting empirical grounding for operational use.

Once validated, extensions may include feedback effects (e.g., how accumulated stress degrades resilience), integration with agent-based or econometric models, or coupling with real-time conflict monitoring systems for early warning applications. Beyond empirical validation, several technical advances could enhance operational possibilities. The framework currently handles static datasets but could be extended for dynamic integration with regularly updated conflict data such as ACLED’s weekly releases, enabling continuous model updates as new events emerge. Real-time implementation would further require feedback mechanisms and potentially a shift toward a plastic plate modeling approach to incorporate evolving ground conditions, news reports, and changing social indicators that may alter material properties between longer data collection cycles. Machine learning approaches could automate parameter calibration by learning from validated humanitarian outcomes, process unstructured news and social media data for early conflict detection, and optimize response function shapes based on observed output–outcome relationships across different contexts.

Robust uncertainty quantification becomes critical for operational deployment, requiring systematic propagation of data quality measures through model outputs to provide confidence intervals around model predictions. This includes developing ML-assisted methods to handle incomplete or delayed conflict reporting, assess data quality automatically, and identify anomalous patterns that may indicate model limitations or emerging conflict dynamics. The framework would also benefit from incorporating user feedback loops where humanitarian practitioners can validate or correct model predictions, enabling continuous learning and parameter refinement in operational settings.

Terminological confusion presents an additional challenge for interdisciplinary communication. The framework’s use of “displacement” to describe physical deformation of the modeled plate conflicts with standard humanitarian terminology where “displacement” refers to forced human migration. While we have attempted to clarify this distinction, the potential for misunderstanding remains significant when communicating with practitioners in conflict-affected settings. Future work should consider alternative terminology that avoids this semantic overlap while maintaining the physical meaning essential to the mathematical framework.

From a methodological perspective, the framework represents one possible application of continuum mechanics principles to social systems. Plate theory provided a structured approach with well-defined material parameters and established solution methods, facilitating the translation between social indicators and physical properties. However, alternative formulations based directly on general continuum analysis could potentially offer greater flexibility in modeling social dynamics without requiring the conceptual adaptations necessary when borrowing from structural engineering. Future research might explore such approaches to determine whether more direct applications of continuum analysis yield insights that are obscured by the constraints of plate-based analogies.

### 4.4. Ethical Considerations

The framework’s apparent objectivity, stemming from its physics-based formulation, risks masking subjective decisions embedded in indicator selection and parameter mapping. These choices reflect assumptions about what constitutes as vulnerability or resilience and may marginalize communities if misapplied. The mathematical sophistication may also create false confidence among users who must understand that while the mathematics are precise, the underlying data and assumptions are not. The model represents a heuristic framework for exploring complexity, not a prediction engine for humanitarian outcomes.

Labeling regions as “fragile” or “at-risk” based on model outputs poses significant risks, particularly when used for resource allocation or policy decisions. Such classifications can have harmful political or social consequences if not accompanied by transparent interpretation and meaningful local consultation. Communities may face stigmatization or become targets for unwanted interventions based on algorithmic assessments that fail to capture local strengths, coping mechanisms, or self-determination preferences.

Data sovereignty presents another critical concern, as social indicators often derive from populations who had no input into how their information would be used for conflict modeling. Communities should retain agency over how they are represented and analyzed, yet current data collection paradigms rarely enable such participation. The framework’s vulnerability definitions may reflect researcher perspectives rather than community understandings, potentially creating self-fulfilling prophecies if used to guide interventions.

The risk of technocratic solutions represents a broader systemic concern. Mathematical sophistication may encourage top-down interventions that bypass local knowledge and community-led solutions, potentially undermining existing social networks and traditional coping mechanisms that do not register in formal datasets. If underlying data reflects historical inequities, the model may inadvertently reinforce existing patterns of marginalization rather than highlighting structural injustices that create vulnerability.

Furthermore, governments or other actors could misuse vulnerability mappings to justify surveillance, population control, or targeting of groups identified as “problematic” or “at-risk.” The dual-use potential of such analytical tools demands careful consideration of who has access to outputs and how results are communicated.

Addressing these concerns requires participatory validation processes, transparent communication of uncertainties and limitations, reflexivity in indicator design, and ongoing dialogue with affected communities. The framework should complement rather than replace local knowledge systems and community-based assessment approaches. Most critically, any operational deployment must include robust safeguards against misuse and mechanisms for community oversight of how their data and circumstances are represented in policy-relevant analysis.

### 4.5. Practical Implications

If appropriately validated, the framework offers several applications. It could support early warning systems by highlighting regions accumulating stress beyond critical thresholds. Scenario analysis could inform intervention strategies by showing how changes in governance, infrastructure, or connectivity alter potential impact landscapes.

As a diagnostic tool, the displacement surface provides a common metric for comparing the severity of conflict across contexts. Its modular structure allows it to adapt to different countries or crises, while the open-source implementation encourages interdisciplinary collaboration and further development [33].

However, even when fully validated, the framework should complement, rather than replace, traditional assessment methods. It excels at structuring complexity and simulating systemic behavior, but it cannot substitute for local knowledge, qualitative insight, or direct field observation.

## 5. Conclusions

We introduced a novel physics-based framework that models conflict-affected regions as elastic systems, “social fabrics”, whose material properties are derived from spatially distributed socioeconomic indicators. Conflict events are treated as external forces acting upon this fabric, producing measurable deformations based on the interplay between event characteristics and local structural conditions.

The approach addresses core challenges in conflict analysis by maintaining a clear mathematical separation between external shocks and system properties. This enables the investigation of how identical events yield divergent outcomes depending on local context—something traditional models struggle to capture. By encoding social complexity into interpretable physical parameters, the framework offers a structured, dynamic alternative to conventional correlation-based methods.

Key contributions include: (1) a translation system that maps diverse social indicators into material parameters through context-sensitive response functions; (2) a conflict event mapping methodology that assigns force magnitudes, spatial distributions, and decay profiles based on event type and intensity; and (3) a finite element implementation that generates emergent behaviors, such as spillover, compounding stress, and nonlinear propagation, directly from mathematical principles, rather than through ad hoc assumptions.

Validation tests confirmed the expected physical behavior of the system, and the Nigeria 2018 case study demonstrated the feasibility of integrating real-world conflict data with heterogeneous social indicators. The results showed that impact is not simply additive or radial but shaped by underlying vulnerabilities, reinforcing the value of this model for representing context-sensitive conflict dynamics.

While the framework requires further empirical validation to assess its predictive accuracy, it establishes a robust methodological foundation for physics-informed conflict modeling. Future research should focus on calibrating parameter values using observed humanitarian outcomes, incorporating dynamic or adaptive material properties, and exploring integration with other modeling approaches.

More broadly, the framework opens new directions for interdisciplinary research by connecting engineering principles with social science concerns. It offers a mathematically rigorous but interpretable system for integrating diverse data, supporting early warning, scenario analysis, and impact forecasting. As conflict continues to threaten global stability and development, physics-informed approaches like the one presented here offer promising pathways for advancing both scientific understanding and practical capabilities for protecting vulnerable populations. However, successful implementation requires careful attention to ethical considerations, community engagement, and rigorous empirical validation to ensure that mathematical sophistication translates into meaningful humanitarian impact.

## Data Availability

The social fabric analysis framework code, implemented using widely adopted Python libraries (NumPy [41], SciPy [42], Matplotlib [43], GeoPandas [44], Rasterio, Pandas [45], Plotly [46], Contextily) without additional specialized software, is freely available under the MIT License at https://github.com/felixschwebel/Social-Fabric-Analysis-Framework (accessed on 28 July 2025) and at https://doi.org/10.5281/zenodo.16509938 (accessed on 28 July 2025) to ensure maximum reproducibility. ACLED conflict data is publicly available at https://acleddata.com/ (accessed on 12 February 2025). Social indicator datasets are available from their respective sources as cited in the paper. An extended version of this work is available as a pre-print at https://arxiv.org/abs/2503.02771 (accessed on 4 March 2025).

## Abbreviations

The following abbreviations are used in this manuscript:

ACLED	Armed Conflict Location and Event Data
BRIC	Baseline Resilience Indicators for Communities
CISI	Critical Infrastructure Spatial Index
DROP	Disaster Resilience of Place
FEA	Finite Element Analysis
FEM	Finite Element Method
GDP	Gross Domestic Product
IOM	International Organization for Migration
PPP	Purchasing Power Parity
SPI	Standard Precipitation Index
SSA	Sub-Saharan Africa
UCDP	Uppsala Conflict Data Program
UN	United Nations
UNHCR	United Nations High Commissioner for Refugees
V-Dem	Varieties of Democracy
WASH	Water, Sanitation and Hygiene

## Appendix A. Social Indicator Mapping Parameters

Table A1 provides a comprehensive overview of all the social indicators used in the proof-of-concept application, including their sources, parameters assignments, response functions, and weighting schemes. This enables full reproducibility of the social fabric construction process.

**Table A1:** Summary of social indicator mapping parameters and their translation functions. This table presents the complete set of indicators used to define the material properties in the proof-of-concept implementation. For each indicator, the source, parameter type, response function, corresponding function parameters, and relative weight are shown.

Indicator	Source	Parameter Type	Response Function	Function Parameter(s)	Weight
Thickness *h*					
CISI	[48]	Resilience	Power Law	$\gamma_{r}=0.5$	0.15
SPI Drought	[49]	Vulnerability	Power Law	$\gamma_{v}=1.2$	0.20
SPI Wetness	[49]	Vulnerability	Power Law	$\gamma_{v}=1.2$	0.20
Health Infrastructure	[48]	Resilience	Logarithmic	$\alpha_{r}=2.5$	0.30
Travel Time to Healthcare	[37]	Prerequisite	Linear	$m_{p}=1.0$	–
Dependency Ratio	[50]	Vulnerability	Exponential	$\beta_{v}=0.8$	0.15
Young’s Modulus *E*					
GDP (PPP)	[51]	Resilience	Linear	$m_{r}=1.0$	0.60
Poverty Rate	[52]	Vulnerability	Power Law	$\gamma_{v}=1.5$	0.20
Childhood Poverty	[53]	Vulnerability	Power Law	$\gamma_{v}=0.5$	0.20
Poisson’s Ratio ν					
Population Density	[54]	Vulnerability	Linear	$m_{v}=2.0$	0.60
Road Density	[55]	Resilience	Logarithmic	$\alpha_{r}=2.0$	0.40

## Appendix B. ACLED Event Classification and Distribution

Table A2 and Table A3 detail the distribution of conflict events in Nigeria during 2018, providing essential context for understanding the scope and characteristics of the conflict data used in our proof-of-concept.

**Table A2:** Distribution of ACLED conflict events [56] in Nigeria during 2018. The table presents the frequency of different event types, including only events with geoprecision code 1 and excluding strategic developments (1254 events total).

Event Type	Count	Percentage
Protests	404	32.22%
Violence against civilians	333	26.56%
Battles	270	21.53%
Riots	198	15.79%
Explosions/Remote violence	49	3.91%

**Table A3:** Detailed distribution of ACLED conflict events [56] and associated fatalities in Nigeria during 2018, broken down by event types and sub-event types (geoprecision code 1, without strategic developments; 1254 events total).

Event Type	Sub-Event Type	Count	Fatalities
Violence against civilians	Attack	303	1023
	Abduction/forced disappearance	24	0
	Sexual violence	6	4
Battles	Armed clash	242	1049
	Government regains territory	20	135
	Non-state actor overtakes territory	8	95
Protests	Peaceful protest	375	0
	Protest with intervention	26	0
	Excessive force against protesters	3	2
Explosions/Remote violence	Remote explosive/landmine/IED	7	5
	Air/drone strike	20	150
	Suicide bomb	20	166
	Grenade	1	0
	Shelling/artillery/missile attack	1	10
	Chemical weapon	–	–
Riots	Violent demonstration	106	71
	Mob violence	92	75

## Appendix C. Conflict Event Mapping Parameters

Table A4, Table A5 and Table A6 provide the complete parameter specifications used to translate ACLED conflict event into forces, enabling full reproducibility of the conflict event mapping process.

**Table A4:** Force magnitude parameters for different conflict types. Base magnitudes ($F_{base}$) are shown as absolute values and percentages of the reference event type (Explosions/Remote violence). The fatality scaling balance ($\alpha_{c}$) determines the mix of linear and logarithmic fatality effects, while the temporal decay rate (λ) controls how quickly event impacts diminish. * The protest base magnitude is further adjusted by the V-Dem Liberal Democracy Index [40], where the final magnitude is calculated as $F_{base}\cdot \left(1-V_{dem}\right)$ to account for varying political contexts.

Event Type	$F_{\mathrm{base}}$ [N]	$\alpha_{c}$	λ
Explosions/Remote violence	$1\times 10^{9}$ N (reference)	0.4	0.0064
Battles	$8.0\times 10^{8}$ N (80%)	0.4	0.0064
Violence against civilians	$6.0\times 10^{8}$ N (60%)	0.2	0.0128
Riots	$3.0\times 10^{8}$ N (30%)	0.3	0.0256
Protests	$1\times 10^{8}$ N (10%) *	0.3	0.0256

**Table A5:** Force distribution patterns assigned to different ACLED sub-event types. Point forces represent highly localized impacts, Gaussian distributions capture focused events with limited spread, linear distributions model gradual impact decline, and constant distributions represent uniform effects across an area.

Event Type	Sub-Event Type	Distribution
Battles	Government regains territory	Linear
	Non-state actor overtakes territory	Linear
	Armed clash	Linear
Protests	Excessive force against protesters	Constant
	Protest with intervention	Constant
	Peaceful protest	Constant
Riots	Violent demonstration	Constant
	Mob violence	Constant
Explosions/Remote violence	Chemical weapon	Linear
	Air/drone strike	Gaussian
	Suicide bomb	Gaussian
	Shelling/artillery/missile attack	Linear
	Remote explosive/landmine/IED	Gaussian
	Grenade	Gaussian
Violence against civilians	Sexual violence	Point Force
	Attack	Point Force
	Abduction/forced disappearance	Point Force

**Table A6:** Force radius parameters for different event types. Base radius ($r_{base}$) defines the initial spread extent of impact in meters, with battles covering the largest area and protests/riots the smallest. Violence against civilians uses point forces without radius parameters (indicated by “–”). The expansion fraction ($\beta_{c}$) and rate (μ) control how impact zones grow over time.

Event Type	$r_{\mathrm{base}}$ [m]	$\beta_{c}$	μ
Explosions/Remote violence	10,000	0.5	0.0512
Battles	25,000	0.5	0.0512
Violence against civilians	–	–	–
Riots	5000	0.1	0.3070
Protests	5000	0.1	0.3070
